# Intra-cameral level of ganciclovir gel, 0.15% following topical application for cytomegalovirus anterior segment infection: A pilot study

**DOI:** 10.1371/journal.pone.0191850

**Published:** 2018-01-29

**Authors:** Samanthila Waduthantri, Lei Zhou, Soon-Phaik Chee

**Affiliations:** 1 Singapore Eye Research Institute, Singapore, Singapore; 2 Singapore National Eye Centre, Singapore, Singapore; 3 Department of Ophthalmology, Yong Loo Lin School of Medicine, National University of Singapore, Singapore, Singapore; 4 Ophthalmology and Visual Sciences Academic Clinical Research Program, Duke-NUS Graduate Medical School, Singapore, Singapore; Hospital JP Garrahan, ARGENTINA

## Abstract

**Purpose:**

To investigate the intra-cameral level of ganciclovir following topical application of ganciclovir gel, 0.15% for cytomegalovirus (CMV) anterior segment infection.

**Design:**

Non-randomized, prospective, interventional clinical study.

**Methods:**

Patients with active CMV anterior segment infection seen at Singapore National Eye Centre, confirmed by positive CMV real time PCR (RT-PCR) of the aqueous humor, that had not been treated with any form of ganciclovir in the preceding 1 month were recruited. They were treated with ganciclovir gel, 0.15% 1cc 5 times a day. Following 6 weeks of treatment, CMV load in the aqueous humor was measured using CMV RT-PCR and the ganciclovir drug levels in tears and aqueous humor were measured using high-performance liquid chromatography-mass spectrometry. The clinical features of the disease activity and the central corneal thickness (CCT) were recorded at the baseline and post-treatment.

**Results:**

There were 29 eyes of 29 patients, of which 23 eyes had CMV anterior uveitis and 6 eyes had CMV endotheliitis. At the end of week 6, 26 eyes had undetectable CMV titre in the aqueous humor and no anterior chamber (AC) activity. Two patients had an increased CMV titre and increased AC inflammation. Both of these patients were non-compliant with the treatment. One patient had a reduced CMV titre in the aqueous humor with minimal AC inflammation. The mean ganciclovir concentration in the aqueous humor and the tears were 17.4 ± 30.6 ng/ml and 20,420.9 ± 33,120.8 ng/ml respectively. Mean CCT was 552.2 ± 42.3 microns. There was a weak correlation between the ganciclovir concentration in the aqueous humor and CCT (Spearmen's r = + 0.42, p = 0.025). There was no significant correlation between the ganiclovir concentration in the tears and CCT (Spearmen's r = + 0.39, p = 0.11).

**Conclusion:**

Ganciclovir levels in the aqueous humor was below the 50% inhibitory dose (ID50) for CMV replication, following topical application of the ganciclovir gel, 0.15%.

**Trial registration:**

SingHealth Centralized Institutional Review Board, Singapore; R733/17/2010, ClinicalTrials.gov; NCT01647529.

## Introduction

Ocular cytomegalovirus (CMV) infection is increasingly being implicated as a cause of anterior uveitis (AU) and endotheliitis in immunocompetent patients, which was initially believed to be idiopathic [1–7]. Chee et al. found that 52.2% of the presumed Posner Schlossman syndrome, 41.7% of the presumed Fuchs heterochromic iridocyclitis^4^ and 83.0% of the corneal endotheliitis [2] were positive for CMV in the aqueous humor. Detecting an etiologic agent and early diagnosis in order to institute specific treatment is important in these patients to prevent blinding complications such as glaucomatous optic neuropathy and corneal decompensation [2,3].

Various regimes have been used to manage CMV AU and endotheliitis, including systemic treatment with intravenous and oral ganciclovir, local therapy using repeated intravitreal ganciclovir injections or implants and topical therapy with ganciclovir gel, resulting in varying rates of success [2–9]. Clincial reponse rate in the patients treated with systemic ganciclovir, ganciclovir implant and intravitreal ganciclovir injections was better than that in the patients treated with topical ganciclovir. However, recurrence rate was high with systemic and local ganciclovir treatment, thus requiring long term maintenance therapy [6]. Recurrence rate upon cessation of ganciclovir treatment was also noted to be higher in the patients with CMV AU than that in the patients with CMV endotheliitis [6,9].

Currently, ganciclovir ophthalmic gel, 0.15% (Laboratories Théa, Clermont-Ferrand Cedex, France) which is intended for the treatment of herpes simplex keratitis, is being used off-label in Singapore to treat CMV anterior segment infection. It is considered an inexpensive alternative with good tolerence and less relapses when compared to other forms of ganciclovir treatment [6]. The recommended dosage is application of 1cc gel 5 times a day. A prevously published study conducted in Singapore showed that 76.6% of the eyes with CMV AU responded well to the topical treatment with ganciclovir ophathalmic gel, 0.15% [6]. On the other hand, other centers reported good experience with guttae ganciclovir, 2% in CMV endotheliits patients [9,10]. Currently, guttae ganciclovir is not commercially available in Singapore.

Pre-clinical studies on rabbit eyes had shown good intra-cameral penetration following topical application of ganciclovir ophthalmic gel in varying concentrations, 0.05%, 0.15% and 0.2% [11–13]. This may vary in humans as the penetration of the topically applied gel into the deep corneal tissue and the anterior and posterior ocular chambers is constrained by the tear flow, complex structure of the cornea, aqueous humor drainage, blood ocular barrier and the chemical stability of the aqueous solution [14,15]. This study aims to investigate the level of ganciclovir in the aqueous humor following topical application of ganciclovir gel, 0.15% and to determine whether the drug penetration correlates with the corneal thickness.

## Methods

### Participants and sample size

We recruited consecutive patients diagnosed with CMV anterior segment infection, either uveitis or endotheliitis at the Singapore National Eye Centre from July 2012 to July 2013. There was no sample size calculation as this was a pilot study.

### Design

This was a non-randomized, single group, prospective interventional study with no comparison group.

### Ethics

Written informed consent was obtained from all the participating subjects. The study was approved by the SingHealth Centralized Institutional Review Board (Protocol number: R733/17/2010). This trial was carried out in accordance with the tenets of the Helsinki declaration.

### Inclusion criteria

We included the subjects aged between 21 and 99 years, diagnosed with CMV anterior segment infection, either AU or endotheliitis based on clinical manifestations and positive CMV real time PCR (RT-PCR) [3] of the aqueous humor. In cases of AU, only the eyes that had keratic precipitates (KPs) on the corneal endothelium and anterior chamber (AC) cells ≥1+ were included. Similarly, in cases of endotheliitis, only the eyes with corneal edema, KPs and AC cells ≥1+ were included.

### Exclusion criteria

We excluded those with other causes of hypertensive AU and endotheliitis such as Herpes simplex virus (HSV) and Varicella-zoster virus (VZV), confirmed by simultaneous PCR analysis [2] of the aqueous humor. Eyes with CMV posterior segment infection were excluded. We also excluded the patients who had been on any form of topical, local or systemic ganciclovir therapy in the preceding one month and those who required systemic ganciclovir therapy or intravitreal ganciclovir injections or implants.

### Intervention

After written informed consent, eligible patients were prescribed topical application of Virgan® ganciclovir ophthalmic gel, 0.15% (Laboratories Théa, Clermont-Ferrand Cedex, France)1cc 5 times a day, which worked out to be once every 3.5 hours, for 6 weeks. This is the only form of topical ganciclovir that is commercially available in Singapore. All the patients were instructed how to use the medication and asked to keep a drug diary to monitor their compliance.

### Outcome measures

Primary outcomes were mean ganciclovir concentration in the aqueous humor and tears and its correlation to the central corneal thickness (CCT), following 6 weeks of topical application of the ganciclovir gel, 0.15%. CCT and the clinical features of the disease activity such as state of the cornea, presence of KPs and AC cells were recorded both at the base line and 6 weeks post treatment. In addition, intraocular pressure (IOP) using Goldmann applanation tonometry and cup to disc ratio (C/D ratio) were also recorded. CCT was measured using Sonogage Corneo-gage™ Plus pachymeter, and an average of five readings were taken. The AC cells and AC activity were graded according to the SUN criteria [16].

At the end of week 6, the patients were reviewed in the clinic, 3.0 hours following the last application of the ganciclovir gel. As the half-life of ganciclovir is 3.5 to 4 hours [17,18], we sampled the aqueous humor 3.0 hours following the last application of the gel. At this study visit, the patients were asked to verbally confirm the gel application time and quantum before proceeding. Tear samples were collected using a capillary tube and sent to analyze ganciclovir drug level. Topical anesthesia was not used prior to collection of the tear samples. Then, the conjunctival sac was irrigated with 100 ml of normal saline to completely wash out any residual drug, prior to collection of the aqueous humor samples. Tetracaine hydrochloride, 1% was instilled for topical anesthesia and an aqueous humor sample of 0.2 ml was withdrawn using a 30 gauge needle under aseptic technique. Of this, 0.1 ml of aqueous humor was sent for CMV RT-PCR analysis and the remaining 0.1 ml of aqueous humor was sent for ganciclovir drug level analysis. Ganciclovir concentration in the aqueous humor and the tear fluid was measured using High-performance liquid chromatography-mass spectrometry (HPLC-MS) [19].

Adverse events (AE) were monitored by the study team throughout the study period. Treatment-related AEs were defined as the AEs for which the causal relationship with the investigational drug could not be ruled out definitively, including the AEs assessed as ‘unlikely related’ to ‘definitely related’ [20]. These included any undesirable symptoms and/or clinical signs.

### Quantitation of ganciclovir in the aqueous humor and tears using liquid chromatography-mass spectrometry (LC-MS/MS) [19]

The concentration of ganciclovir in the aqueous humor and tears was determined by HPLC-MS/MS. Ganciclovir pure standard and HPLC grade ammonium formate were purchased from Sigma-Aldrich (St Louis, MO). Acetonitrile and formic acid were purchased from Merck Chemicals (Darmstadt, Germany). Chromatographic separation was performed by a Waters 2695 Separations Module (Waters, Milford, MA) with a ZIC HILIC column, 2.1 × 100 mm, 3.5 μm, 200 Å (Merck SeQuant AB, Umea, Sweden). The autosampler and column heater temperatures were maintained at 10°C and 30°C, respectively. The mobile phase was: A, 20 mM ammonium formate in 9:1 water/acetonitrile and B, 0.1% formic acid in 9:1 acetonitrile/water. The gradient profile was: 95% B at 0 min to 85% B at 8 min, 40% B from 8.5 to 10 min and 95% B at 10.5 min. The flow rate was 0.2 ml/min. Detection was performed by an API 2000 triple quadruple mass spectrometer (AB Sciex, Concord, Canada) with an ESI source operating in positive ionization mode. The MRM transition 256.1/152.1 was used for quantitation. The retention time of ganciclovir was 4.7 min.

Aqueous humor samples (25 μL) were dried in a vacuum concentrator and reconstituted in 2:1 acetonitrile/water (50 μL). Samples were vortexed, centrifuged and the supernatants similarly analyzed by LC-MS/MS. Tear samples were diluted with 2:1 acetonitrile/water (10 to 1000-fold) and vortexed briefly. Samples were then centrifuged for 10 min at 14000 g (4°C) and the supernatants were analyzed by LC-MS/MS. In both cases, 20 μL of supernatant was injected for LC-MS/MS analysis. The limit of quantitation (LoQ) was estimated at 1 ng/ml (S/N = 10).

### Statistical analysis

Statistical significance was set at p≤0.05. As the data were not normally distributed, non-parametric Spearman rank correlation test was used to evaluate the correlation between CCT and the ganciclovir concentration in aqueous humor and tears. SPSS ver. 17 (IBM, Chicago, IL) was used for analysis.

## Results

**Fig 1:**
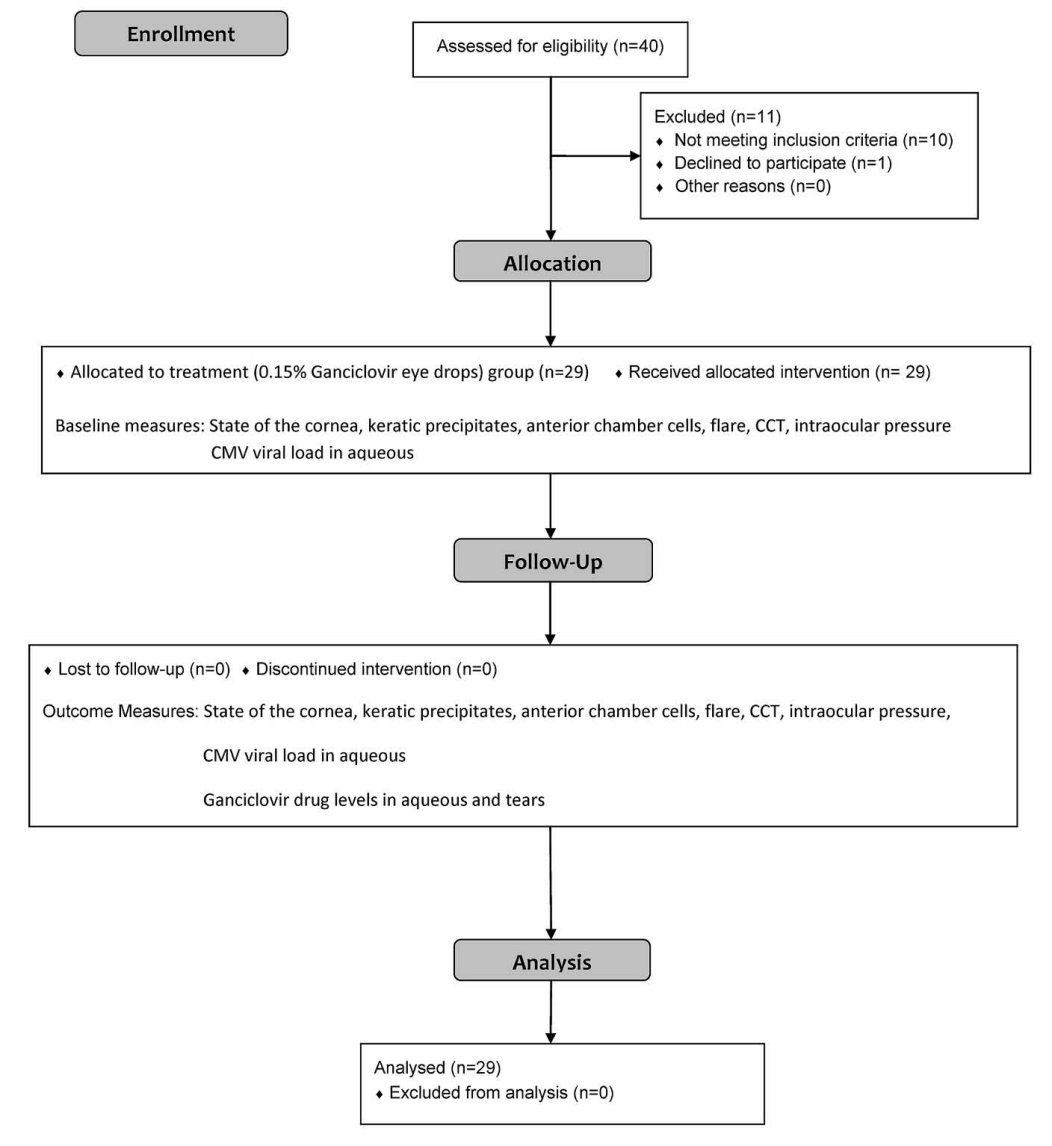
Participants’ flow diagram. (CONSORT 2010 Flow Diagram from Schulz KF, Altman DG, Moher D, for the CONSORT Group. CONSORT 2010 Statement: updated guidelines for reporting parallel group randomised trials. BMJ 2010; 340:c332).

Twenty nine eyes of 29 patients (20 males and 9 females) aged between 32 and 82 years (Mean age 60.4 ± 12.5 years) were recruited (Table 1). Twenty three eyes had CMV AU and six eyes had CMV endotheliitis. All patients completed the 6 weeks of follow-up (Fig 1). Mean CCT was 552.2 ± 42.3 microns. The drug diaries revealed that all but 2 patients were compliant with the study medication.

**Table 1:** Demographics, clinical features and outcomes.

Patient No:	Sex	Age (years)	Diagnosis	Disease activity (Pre-treatment)		Disease activity (Post-treatment)	Real-time PCR (Pre-treatment)	Real-time PCR (Post-treatment)
1	M	76	AU RE	AC cells 1+	Quiet		54,000	Undetectable Tire
2	M	66	AU LE	AC cells 1+	Quiet		3,100	Undetectable Tire
3	M	61	AU RE	AC cells 2+	AC cells 3+		150,000	510,000
4	M	53	AU RE	AC cells 1+	Quiet		13,000	Undetectable Tire
5	F	52	AU LE	AC cells 1+	Quiet		50	Undetectable Tire
6	M	45	AU LE	AC cells 1+	Quiet		59,000	Undetectable Tire
7	M	70	Endotheliitis LE	AC cells 1+	Quiet		11,000	Undetectable Tire
8	M	42	Endotheliitis RE	AC cells 1+	Quiet		150,000	Undetectable Tire
9	M	73	AU LE	AC cells 1+	Quiet		25,800	Undetectable Tire
10	F	82	AU RE	AC cells2+	AC cells 3+		40,000	850,000
11	F	58	AU LE	AC cells 1+	Quiet		4,146	Undetectable Tire
12	M	72	AU RE	AC cells 1+	AC cells 1/2+		293,370	2,225
13	M	63	AU LE	AC cells 1+	Quiet		13,990	Undetectable Tire
14	F	55	AU LE	AC cells 1+	Quiet		110,00	Undetectable Tire
15	M	59	AU LE	AC cells 1+	Quiet		5,760	Undetectable Tire
16	F	66	AU LE	AC cells 1+	Quiet		947	Undetectable Tire
17	M	75	Endotheliitis LE	AC cells 2+	Quiet		128,829	Undetectable Tire
18	M	39	AU RE	AC cells 1+	Quiet		29,951	Undetectable Tire
19	F	63	AU RE	AC cells 1+	Quiet		136,86	Undetectable Tire
20	M	58	AU LE	AC cells 1+	Quiet		690	Undetectable Tire
21	M	53	AU LE	AC cells 1+	Quiet		4,716	Undetectable Tire
22	M	65	Endotheliitis LE	AC cells 1+	Quiet		196,306	Undetectable Tire
23	M	50	AU RE	AC cells 1+	Quiet		633	Undetectable Tire
24	M	69	AU RE	AC cells 1+	Quiet		4,785	Undetectable Tire
25	M	54	Endotheliitis LE	AC cells 1+	Quiet		439,680	Undetectable Tire
26	F	32	AU RE	AC cells 1+	Quiet		1,960	Undetectable Tire
27	M	80	Endotheliitis LE	AC cells 1+	Quiet		2,548	Undetectable Tire
28	F	63	AU RE	AC cells 1+	Quiet		460	Undetectable Tire
29	F	46	AU RE	AC cells 1+	Quiet		1,824	Undetectable Tire

Right eye (RE) Left eye(LE)

**Fig 2:**
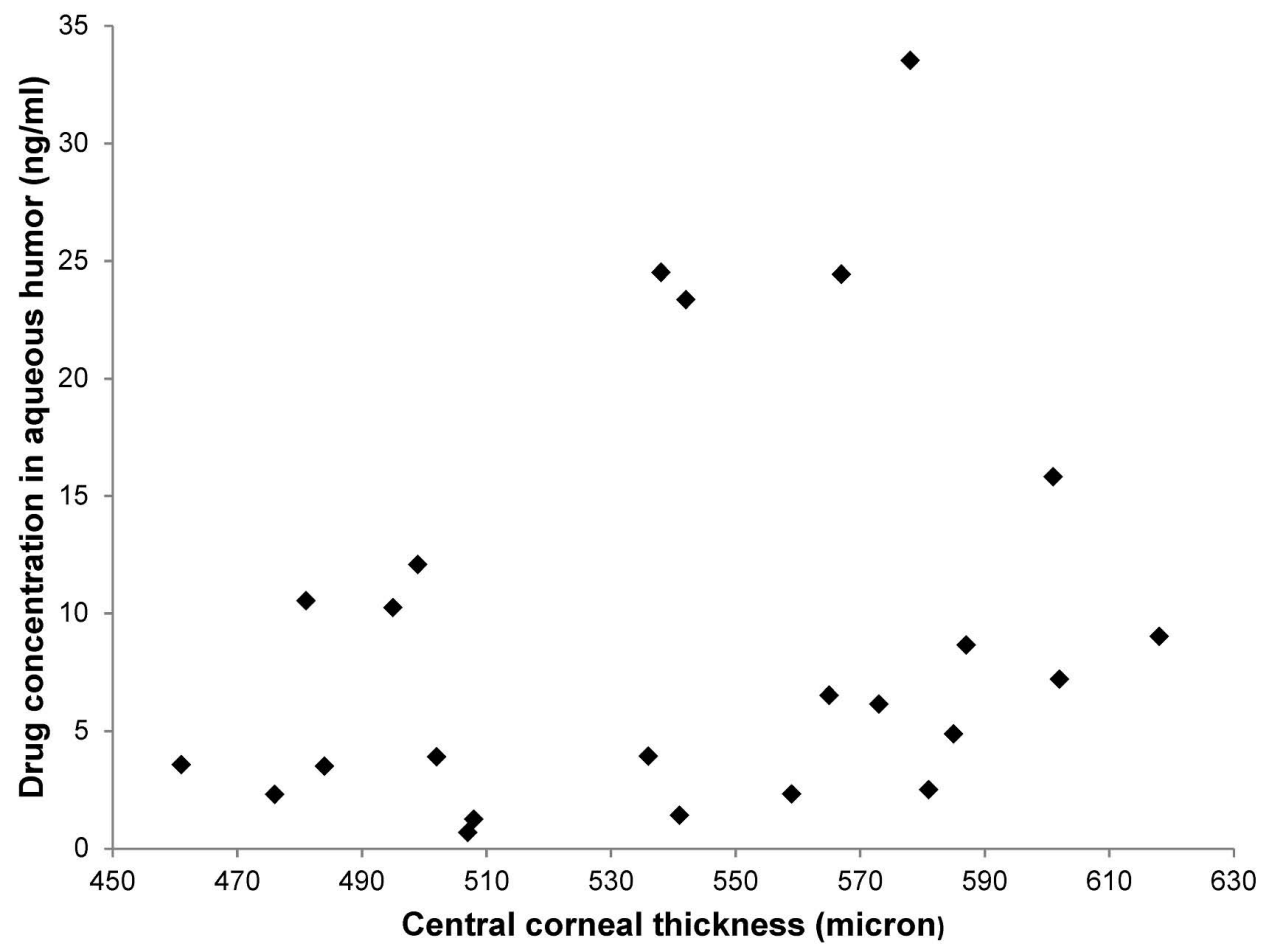
Ganciclovir concentration in aqueous humor.

**Fig 3:**
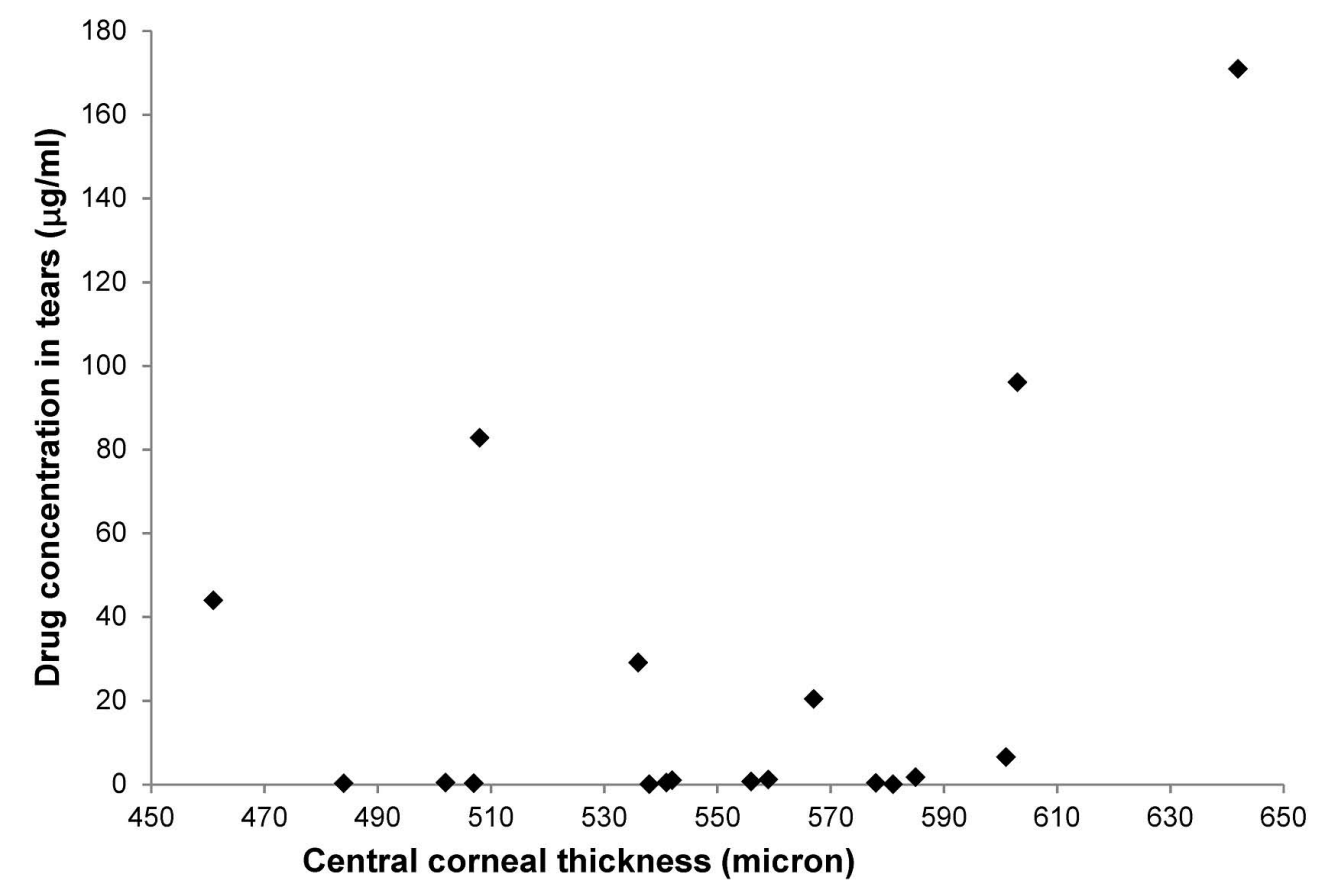
Ganciclovir concentration in tears.

The mean concentrations of ganciclovir in the aqueous humor and tears were 17.4 ± 30.6 ng/ml and 20,420.9 ± 33,120.8 ng/ml respectively. The ganciclovir drug levels in the aqueous humor and tear samples are shown in Figs 2 & 3 respectively. Ganciclovir concentration in one of the aqueous humor samples was below the LoQ (0.68 ng/ml). Ganciclovir concentrations in four aqueous humor samples were between 1 ng/ml and 3 ng/ml (1.3, 1.4, 2.3 and 2.4 ng/ml). There was a weak correlation between the ganciclovir drug levels in the aqueous humor and CCT (Spearmen's r = +0.42, p = 0.025). There was no significant correlation between the ganciclovir drug levels in the tears and CCT (Spearmen's r = +0.39, p = 0.11). At the end of week 6, 26 patients (89.7%) had undetectable CMV titres in the aqueous humor. One patient with AU had a reduced CMV titre (2,225 viral DNA copies/ml from a baseline of 293,370 viral DNA copies/ml) in the aqueous humor with ½+ AC cells and normal IOP after 6 weeks of treatment (Table 1). Two patients with AU had increased CMV titres in the aqueous humor (850, 000 viral DNA copies/ml and 510,000 viral DNA copies/ml from baseline levels of 40,000 viral DNA copies/ml and 150,000 viral DNA copies/ml respectively) with raised IOP and increased AC activity at the end of week 6. These values belonged to the two patients who were found to be non-compliant to the study medication and had incompletely filled diaries, reporting that the frequency of the instillation regimen was excessive for their busy schedules.

None of the patients developed any side-effects nor adverse events from using the medication. Neither were there any serious adverse events reported in this study. We did not perform subgroup analysis to compare the ganciclovir concentrations in the aqueous humor and tear samples between AU and endotheliitis eyes due to small sample sizes in each subgroup.

## Discussion

In this study, topical application of ganciclovir ophthalmic gel, 0.15% exhibited a low intra-cameral drug level with a mean aqueous humor concentration of 17.4 ± 30.6 ng/ml, falling severely short of the 50% inhibitory dose (ID50) for CMV replication. ID50 for CMV replication is 250 ng/ml [21]. As seen in Fig 2, the ganciclovir concentrations in several aqueous humor samples were close to LoQ. The reader should note that the measurements close to LoQ (recovery% = 80.1%) are generally less accurate than those in the mid-range of the calibration curve (recovery% = 86.0%~108.0% for different concentrations). Ganciclovir concentration in one of the aqueous humor samples was below 1ng/ml (0.68ng/ml). This reading was excluded from the analysis as this measurement was out of the range of the calibration curve. Ganciclovir concentrations in four aqueous humor samples were between 1ng/ ml and 3ng/ml (1.3, 1.4, 2.3 and 2.4 ng/ml). We found that inclusion or exclusion of these measurements in the analyses did not affect the conclusion of the analyses.

Ganciclovir drug levels in both aqueous humor and tear samples were variable. There was only a weak correlation between the ganciclovir concentration in the aqueous humor and the CCT. Despite these findings, there was clearance of CMV from the aqueous humor following continuous application of ganciclovir gel for 6 weeks with good tolerance.

### Comparison with previous studies

Ocular pharmacokinetics studies in rabbit eyes have shown a rapid and relevant penetration of ganciclovir into the cornea and the anterior segment of the eye. Intra-cameral levels of ganciclovir in rabbit eyes reached a concentration of 394 ± 419 ng/ml following topical application of ganciclovir ophthalmic gel, 0.2% 4 times a day for 12 days [11–13]. Ganciclovir concentrations (Cmax) in the cornea, conjunctiva, aqueous humor and iris/ciliary body remained above ID50 for CMV replication for more than 4 hours. In comparison, concentrations of ganciclovir in the aqueous humor were extremely low, ranging from 0.67 ng/ml to 33.53 ng/ml in this human study. Of note, the drug concentration used in our study was lower than that in the rabbit study. We had confirmed the gel application time and quantum before sampling of tears and aqueous humor and also excluded the results of the two non-compliant patients to ensure the reliability of these results.

Ganciclovir has a poor ocular bioavailability as a result of its relatively low partition coefficient [22]. The diffusion of this drug from the cornea to aqueous humor may further depend on the overall integrity of the corneal structures, which is probably compromised in the CMV infected eyes [11–15,23–27]. Corneal epithelium presents as a continuous layer of plasma membrane to the tear film, largely resisting the penetration of the hydrophilic drugs. On the other hand, corneal stroma serves as a major ocular depot for the topically applied hydrophilic drugs [28]. CMV infection can cause corneal endothelial cell loss, and a high CMV viral load in the aqueous humor is associated with an increased corneal endothelial cell loss [23]. Impairment of the corneal endothelial cell layer can result in localized or diffuse stromal edema [26, 27]. Therefore, ganciclovir gel which has a hydrophilic polymer base may preferentially concentrate in the corneal stroma of the eyes with CMV endotheliitis, partly accounting for the reduced intra-cameral drug levels.

A recent study on seven patients with CMV endothellitis reported a mean ganciclovir concentration of 162.0±202.4 ng/ml in the aqueous humor following topical application of ganciclovir gel, 0.15% six times a day for 12 weeks [29]. The long duration and the high frequency of application of the drug may have resulted in higher concentrations of ganciclovir in the aqueous humor as compared to our study. However, the ganciclovir concentrations remain below ID50 for CMV replication. Indeed, re-emergence of CMV without symptoms was observed in one eye which had a low aqueous drug level.

In rabbits, ganciclovir concentration was markedly higher in the solid ocular tissues than that in the ocular fluids [13]. A histopathological study on immunocompromised cadaveric eyes found cytomegalic inclusion bodies predominantly in the iris, ciliary body and endothelial cells of the Schlemm's canal [30]. Ganciclovir has a poor water solubility and a high affinity for the melanin in the cells [13,31], which partly accounts for the drug’s preferential distribution in the solid ocular tissues such as iris, as compared with aqueous humor. This may explain its therapeutic effect despite the low aqueous drug levels, as evidenced by the findings in human studies. Interestingly, frequent manifestations of the CMV anterior segment infection include the presence of fresh pigmented KPs and iris depigmentation [3], targeting the same melanin containing cells within which the ganciclovir concentrates. This perhaps enhances its therapeutic effect. In addition, ganciclovir has been found to preferentially accumulate in host cells infected with virus rather than healthy cells [32,33]. It has an intracellular half-life of more than 24 hours [34], further explaining the effective clearance of CMV in our study patients despite apparent sub therapeutic ganciclovir concentrations in the aqueous humor. For these reasons, we believe that the aqueous humor ganciclovir levels in healthy eyes may not be directly comparable to CMV infected eyes.

In a previously published study on healthy human eyes, there was a wide range of inter-individual and intra-individual variation in the ganciclovir concentration in tears, ranging from 0.92 to 6.86 μg/ml. These concentrations were measured 2 hours and 45 minutes after 4 instillations of ganciclovir gel, 0.15% [33]. In our study, ganciclovir concentration in the tears ranged from 0.03 to170.83 μg/ml after instillation of the study medication 5 times a day for 6 weeks and sampling the tears 3.0 hours after the last application. This wide range in concentration is likely due to variation in tear turn-over rate among individuals and induced reflex tearing that occurs during tear collection [33].

Similar to other studies [6,29,35–37], our patients demonstrated good tolerance to ganciclovir ophthalmic gel, 0.15% with minimal toxicity and complications. However, intensive use of ganciclovir gel for prolonged periods of time may incur epithelial toxicity. Nonetheless, this rarely causes patients to discontinue its use.

## Clinical significance and conclusions

Ganciclovir concentrations in the aqueous humor were below the ID50 for CMV replication following topical application of the ganciclovir gel, 0.15%. There was a weak correlation between the aqueous humor drug levels and CCT. The preferential concentration of the drug in the solid ocular tissue in CMV infected eyes [13,30–33] may explain the clinical improvement seen in these eyes. Due to the short half-life of the drug, the frequency of instillation required from the patients resulted in a non-compliance rate of 6.9%. Patients were able to tolerate this form of ganciclovir therapy well.

Hence, the topical ganciclovir gel, 0.15% can be considered a well-tolerated and inexpensive alternative to other forms of ganciclovir therapy for CMV anterior segment infection. Further research into improving the pharmacokinetics of ganciclovir is needed to improve the efficacy of the topical therapy which carries significant advantages over oral administration of the drug.

## Supporting information

S1 File Study protocol. (PDF)

S2 File Trend check list. (DOC)
