# Supplementary material for: Intra-cameral level of ganciclovir gel, 0.15% following topical application for cytomegalovirus anterior segment infection: A pilot study
Source: PLoS One. 2018 Jan 29;13(1):e0191850. doi: 10.1371/journal.pone.0191850 (PMC5788360; doi:10.1371/journal.pone.0191850)
Supplement: S2 File — (DOC) [file pone.0191850.s002.doc]

# RESEARCH STUDY PROTOCOL

| Protocol Title | : | Intra-cameral penetration of ganciclovir following topical administration of 0.15% ganciclovir gel (VIRGAN©) for CMV anterior uveitis / endotheliitis |
| --- | --- | --- |
| Protocol Number: | : | **R733/17/2010** |
| Version No/ Date | : | **Version 3 dated 24 November 2011** |

**Study Team**

| **Contact details of Principal Investigator** | | |
| --- | --- | --- |
| Name | : | Chee Soon Phaik |
| Designation | : | Head, Senior Consultant |
| Department | : | Ocular Inflammation and Immunology |
| Institution | : | Singapore National Eye Centre |
| Telephone | : | 63228888 |

| **Contact details of co-Investigator** | | |
| --- | --- | --- |
| Name |  | Samanthila Waduthantri |
| Designation |  | Clinical research fellow |
| Institution |  | Singapore National Eye Centre |
| Telephone |  | 90391779 |

| **Contact details of Collaborators** | | |
| --- | --- | --- |
| Name | : | Gemmy Cheung |
| Designation | : | Consultant |
| Institution | : | Singapore National Eye Centre |
| Telephone | : |  |

| Name | : | Zhou Lei |
| --- | --- | --- |
| Designation | : | Principal Research Scientist |
| Institution | : | Singapore Eye Research Institute |
| Telephone | : |  |
| Name | : | Alireza Hedayatfar |
| Designation | : | Singapore National Eye Centre |
| Institution | : | International Fellow |
| Name | : |  |
| Designation | : |  |
| Institution | : |  |

**Principal Investigator’s Experience**

The principal investigator has experience in a wide range of clinical research and laboratory techniques, and has worked with Cytomegalovirus (CMV) uveitis / endotheliitis patients for past ten years.

**Study Background**

This study will be conducted in compliance with the protocol, SGCP and the applicable regulatory requirement(s).

There is a high prevalence of Cytomegalovirus (CMV) sero-positivity of 87% in our general population (5). Cytomegalovirus ocular infection is well documented in the form of CMV retinitis in immunocompromised individuals. However, it is increasingly being implicated as a cause of anterior uveitis / endotheliitis in immunocompetent patients and has been identified recently as one of the disease entities which was previously recognized as idiopathic anterior uveitis / endotheliitis. We found CMV DNA on Tetraplex PCR in 22.8% of our eyes with hypertensive anterior uveitis in immnocompetant patients (5).

Cytomegalovirus anterior uveitis may present as a mild self-limiting iritis, to an acute anterior uveitis, with recurrent episodic iritis and raised intraocular pressure resembling Posner Schlossman Syndrome (PSS), or a more chronic and severe form resembling Fuchs Heterochromic Iritis (FHI) and even corneal endotheliitis (1-11). In a previous study we found 52.2% of patients with presumed PSS, 41.7% of patients with presumed FHI and 83% of patients with corneal endotheliitis to be CMV-positive on aqueous Tetraplex PCR (5). Early diagnosis and treatment is important in these patients to prevent severe complications such as intractable glaucoma and corneal decompensation (5).

Various regimes are used in the literature to treat CMV anterior uveitis/endotheliitis including systemic treatment with intravenous and oral ganciclovir, local therapy with repeated intravitreal injections or intravitreal ganciclovir implants, and topical therapy with ganciclovir gel with varying degrees of success (3, 5, 12-14). Ganciclovir, dihydroxypropoxymethylguanine (DHPG) is a guanosine derivative that acts by competitive inhibition of viral DNA polymerase and by direct incorporation into viral DNA. The consensus is that upon stopping the ganciclovir treatment, some patients have immediate relapses thus requiring a long-term maintenance therapy in these patients. Long-term ganciclovir therapy has significant side effects and is costly. Systemic ganciclovir carries a risk of bone marrow suppression and renal and liver dysfunction that needs frequent monitoring. Intravenous therapy requires hospitalization. Oral valganciclovir though as effective, is expensive, costing S$11,000 for 1 month of induction therapy and 2 months of maintenance therapy. Local therapy with repeated intravitreal ganciclovir injections carries a risk of endophthalmitis and retinal detachment. Intravitreal ganciclovir slow release implants carries similar risks and costs US$17,000 per implant. Hence topical ganciclovir treatment is a promising cheaper alternative with less systemic side effects and recurrence compared to systemic and local therapy.

Ganciclovir ophthalmic gel, 0.15% (Virgan®; Laboratories Théa) is a transparent aqueous gel with a hydrophilic polymer base. It has been used to treat herpetic viral infections for more than 10 years. In SNEC, we have used oc. virgan off- label, effectively with good tolerance as a long-term maintenance therapy to prevent recurrences in patients with CMV related anterior segment disease, that are unable to afford or tolerate other forms of therapy (paper is currently in press, British Journal of Ophthalmology).

The gel formulation allows for more prolonged contact time with the eye than an oil-based formulation. It has a similar tonicity to human tears and both its pH (7.45) and osmolarity (300 mOsmol) were adjusted to values close to normal physiologic values. It has a long and stable shelf life and has demonstrated good tolerability. Previous studies have shown its use topically has minimal local and systemic toxicity.

Ocular pharmacokinetics studies in rabbits have shown a rapid and relevant penetration of ganciclovir into the cornea. Studies shows, intracameral penetration of ganciclovir in rabbit corneas reaches a concentration of 394±419ng/ml, after the application of 0.2% ganciclovir ophthalmic gel four times a day for 12 days. This level was obtained four hours after the final drug application on the day 12 (16). The minimum concentration of ganciclovir that inhibits CMV replication (IC50) is 250ng/ml (17).

However, the intracameral pharmacokinetics of topical ganciclovir 0.15% gel is not known in humans. Penetration into the deeper corneal tissue and anterior and posterior ocular chambers following topical application is constrained by tear flow, complex structure of the cornea, aqueous humor drainage, blood ocular barriers and chemical stability in aqueous solutions.

A proper understanding of intracameral penetration of 0.15% ganciclovir gel (VIRGAN©) following topical administration would allow us to determine objectively, if and when topical ganciclovir 0.15% is able to reach the minimum levels required to inhibit CMV, and correlate these levels with clinical response and corneal thickness. This will be helpful in determining the efficacy, correct dosage, frequency and the duration of the topical ganciclovir therapy for CMV anterior uveitis and endotheliitis.

# **Clinical importance**

This study will help clinicians to understand the intracameral penetration of 0.15% ganciclovir gel (VIRGAN©) following topical administration.

A proper understanding of the intracameral concentration of 0.15% ganciclovir gel (VIRGAN©) following topical administration would allow clinicians to determine objectively, if and when topical ganciclovir 0.15% is able to reach the minimum levels required to inhibit CMV, and correlate these levels with clinical response and corneal thickness. This will be helpful in determining the efficacy, correct dosage, frequency and the duration of the topical ganciclovir therapy for CMV anterior uveitis/endotheliitis.

# **Study Objectives and Purpose**

1. To investigate the intra-cameral level of ganciclovir following topical application of 0.15% ganciclovir gel (VIRGAN©) for Cytomegalovirus (CMV) anterior uveitis and endotheliitis.

2. To investigate if the intra-cameral drug level correlates with the central corneal thickness

## Study Design

**Study Design:**

Prospective, cross-sectional, interventional clinical study

**Methods:**

Thirty patients who are diagnosed with Cytomegalovirus (CMV) anterior segment infection, either uveitis or endotheliitis, at the investigators clinic in Singapore National Eye Centre, who have a positive aqeous real time PCR (RT-PCR) and/or positive tetraplex PCR for CMV and have not had any form of ganciclovir treatment in the past 1 month, will be recruited in the study after taking an informed consent. This will also include the patients who have never been treated with antiviral therapy and those with recurrent disease and have not had any form of gancyclovir treatment for past 1 month.

The clinical features of the active disease will be recorded in the data collection sheet and will include, documention of the state of the cornea, KPs, AC cells, flare, flare meter reading (where possible),central corneal thickness, Intra ocular preassure(IOP) and C:D ratio.

All the patients will be started on 0.15% oc.Virgan, 1cc 5 times a day.

Following 6 weeks of continuous application of Virgan gel 0.15% 1cc 5 times a day, the patient will be reviewed at the clinic within 3 hours following the last application of the gel.

1. Clinical features mentioned above will be documented for activity assessment

2. Central corneal pachymetry will be done. An average of 5 readings will be taken using Sonogage CORNEO-GAGE™ PLUS pachometer.

3. Tears will be collected using a capillary tube

4. Following irrigation of the conjunctival sac with 100ml of N/S to wash out any residual drug, aseptic technique is practised before an aqueous sample 0.2ml is drawn. 0.1ml will be sent for RT-PCR for CMV status and another 0.1ml will be sent for ganciclovir drug level by HPLC method.

All the patients will be asked to keep a drug diary to monitor the compliance.

**Analysis:**

Ganciclovir concentration in aqueous humor and tear fluid will be measured using liquid chromatography-mass spectrometry (LC-MS). A very sensitive method based on LC-MS was established in a prevous pilot study using 17 clinical samples (aqueous humor or vitreous after different treatments, i.e. topical, oral, or intravitreal).

Solid phase extraction (SPE) will be used to remove the matrix (proteins, etc.) in aqueous humor and tear fluid. The LC-MS analysis will be performed with Waters 2690 equipped with a Photodiode Array Detector (Waters Associates, U.S.A.) and a Micromass Mass Spectrometer (Micromass U.K.). The separation column will be a RP8, 3.5 μm, 4.6 x 75 mm. In order to get good separation of ganciclovir with other components in aqueous humor, the column will be eluted by 5%A / 95%B (A: 0.02%TFA + 0.1%HAc in water; B: 0.02%TFA + 0.1%HAc in acetontrile) and increased linearly to 50%A / 50%B in 30 minutes at the flow rate of 0.2 ml/min. The electrospray positive mode will be employed for monitoring the protonated ion of ganciclovir. All mass spectra will be recorded under a full scan operation for positive ions, with a scan range from m/z 140 to 500. The quantification will be carried out with the selected-ion recording (SIR) mode by monitoring its protonated ion (m/z = 256.2). For quantitation, calibration curve will be established in the concentration range of 0.03 ng/μl to 1.5 ng/μl.

**Expected findings:**

1.Median concentration of ganciclovir in anterior chamber and ocular surface.

2. Correlation of intracameral concentration of virgan with the corneal thickness, as some of the patients might have a thickened corneas secondary to CMV infection.

**Inclusion criteria:**

# Age between 21 and above

# Patients who are diagnosed with anterior uveitis or endotheliitis with a positive aqueous real time PCR (RT-PCR) and/or positive tetraplex PCR for Cytomegalovirus (CMV)

# Patients with relapses and recurrent anterior segment disease that is PCR positive for CMV in aqueous

# Have not been on VIRGAN or any other form of ganciclovir therapy for the past 1 month

# Consent to undergo anterior chamber tap and give aqueous and tear samples for the study

# Able to undergo relevant tests (corneal pachymetry and laser flare cell photometry)

- Able to come for subsequent follow-up visits

**Exclusion criteria:**

- CMV anterior uveitis with associated retinitis
- Other causes of hypertensive anterior uveitis / endotheliitis such as HSV, VZV infection
- Patients who have been on any form of (topical, local or systemic) ganciclovir therapy for the past1 month.
- Patients who are allergic to ganciclovir
- Patients who will require systemic or intra-vitreal ganciclovir therapy
- Immunocompromised patients
- Positive for HIV, Hep B and Hep C
- Not keen on participating in the study
- Patients who are incapable, either by law or mental state, of giving consents in their own right.
- Patients who are either unable or unwilling to keep scheduled appointments and adhere to the other aspects of the protocol
- Patients who are pregnant or breastfeeding
- Any other specified reason as determined by the clinical investigator.

### Study Timeline

| **STUDY TIMELINE *** | | | | | | | | | | | | | | | | | | | | | | | | | | | |
| --- | --- | --- | --- | --- | --- | --- | --- | --- | --- | --- | --- | --- | --- | --- | --- | --- | --- | --- | --- | --- | --- | --- | --- | --- | --- | --- | --- |
| TRIAL ACTIVITY | | Date | | Year 2010 | | | | | | | | | | | | | | | | | | | | | | | |
|  |  |  |  | Jan | | Feb | | Mar | | Apr | | May | | June | | July | | Aug | | Sept | | Oct | | Nov | | Dec | |
|  |  | From | To |  |  |  |  |  |  |  |  |  |  |  |  |  |  |  |  |  |  |  |  |  |  |  |  |
| Study Initiation | Plan | Aug2010 | - |  |  |  |  |  |  |  |  |  |  |  |  |  |  | ● |  |  |  |  |  |  |  |  |  |
|  | Actual |  |  |  |  |  |  |  |  |  |  |  |  |  |  |  |  |  |  |  |  |  |  |  |  |  |  |
| Recruitment | Plan | Aug2010 | Feb2011 |  |  |  |  |  |  |  |  |  |  |  |  |  |  |  | ● | ● | ● | ● | ● | ● | ● | ● | ● |
|  | Actual |  |  |  |  |  |  |  |  |  |  |  |  |  |  |  |  |  |  |  |  |  |  |  |  |  |  |
| Follow-up Visits | Plan | Oct 2010 | May 2011 |  |  |  |  |  |  |  |  |  |  |  |  |  |  |  |  |  |  | ● | ● | ● | ● | ● | ● |
|  | Actual |  |  |  |  |  |  |  |  |  |  |  |  |  |  |  |  |  |  |  |  |  |  |  |  |  |  |
| Interim analysis (if applicable) | Plan | Nov 2010 |  |  |  |  |  |  |  |  |  |  |  |  |  |  |  |  |  |  |  |  | ● | ● |  |  |  |
|  | Actual |  |  |  |  |  |  |  |  |  |  |  |  |  |  |  |  |  |  |  |  |  |  |  |  |  |  |
| Study Closure | Plan | NA |  |  |  |  |  |  |  |  |  |  |  |  |  |  |  |  |  |  |  |  |  |  |  |  |  |
|  | Actual |  |  |  |  |  |  |  |  |  |  |  |  |  |  |  |  |  |  |  |  |  |  |  |  |  |  |
| Final report | Plan | June  2011 | - |  |  |  |  |  |  |  |  |  |  |  |  |  |  |  |  |  |  |  |  |  |  |  |  |
|  | Actual |  |  |  |  |  |  |  |  |  |  |  |  |  |  |  |  |  |  |  |  |  |  |  |  |  |  |

*****: To amend as per study requirement

### Timeline

| **STUDY TIMELINE *** | | | | | | | | | | | | | | | | | | | | | | | | | | | |
| --- | --- | --- | --- | --- | --- | --- | --- | --- | --- | --- | --- | --- | --- | --- | --- | --- | --- | --- | --- | --- | --- | --- | --- | --- | --- | --- | --- |
| TRIAL ACTIVITY | | Date | | Year 2011 | | | | | | | | | | | | | | | | | | | | | | | |
|  |  |  |  | Jan | | Feb | | Mar | | Apr | | May | | June | | July | | Aug | | Sept | | Oct | | Nov | | Dec | |
|  |  | From | To |  |  |  |  |  |  |  |  |  |  |  |  |  |  |  |  |  |  |  |  |  |  |  |  |
| Study Initiation | Plan | Aug2010 | - |  |  |  |  |  |  |  |  |  |  |  |  |  |  |  |  |  |  |  |  |  |  |  |  |
|  | Actual |  |  |  |  |  |  |  |  |  |  |  |  |  |  |  |  |  |  |  |  |  |  |  |  |  |  |
| Recruitment | Plan | Aug2010 | Feb 2010 | ● | ● | ● |  |  |  |  |  |  |  |  |  |  |  |  |  |  |  |  |  |  |  |  |  |
|  | Actual |  |  |  |  |  |  |  |  |  |  |  |  |  |  |  |  |  |  |  |  |  |  |  |  |  |  |
| Follow-up Visits | Plan | Oct 2011 | May 2011 | ● | ● | ● | ● | ● | ● | ● | ● | ● |  |  |  |  |  |  |  |  |  |  |  |  |  |  |  |
|  | Actual |  |  |  |  |  |  |  |  |  |  |  |  |  |  |  |  |  |  |  |  |  |  |  |  |  |  |
| Interim analysis (if applicable) | Plan | Nov 2010 |  |  |  |  |  |  |  |  |  |  |  |  |  |  |  |  |  |  |  |  |  |  |  |  |  |
|  | Actual |  |  |  |  |  |  |  |  |  |  |  |  |  |  |  |  |  |  |  |  |  |  |  |  |  |  |
| Study Closure | Plan | NA |  |  |  |  |  |  |  |  |  |  |  |  |  |  |  |  |  |  |  |  |  |  |  |  |  |
|  | Actual |  |  |  |  |  |  |  |  |  |  |  |  |  |  |  |  |  |  |  |  |  |  |  |  |  |  |
| Final report | Plan | June  2011 | - |  |  |  |  |  |  |  |  |  |  |  |  |  |  |  |  |  |  |  |  |  |  |  |  |
|  | Actual |  |  |  |  |  |  |  |  |  |  |  |  | ● |  |  |  |  |  |  |  |  |  |  |  |  |  |

*****: To amend as per study requirement

### Direct Access to Source Data

The Investigator(s)/ Singapore National Eye Centre will permit study-related monitoring audits, MCRC and or EC review and regulatory inspection(s), providing direct access to source data/ document.

## Ethics

This study will be conducted in accordance with the ethical principles that have their origin in the Declaration of Helsinki and that are consistent with the Singapore Good Clinical Practice and the applicable regulatory requirements.

The final study protocol, including the final version of the Patient Information and Informed Consent Form, must be approved or given a favourable opinion in writing by the SERI Institutional Review Board and regulatory approval from the Health Sciences Authority, HSA (regulatory approval only applicable for drug-related clinical trials), prior to the enrolment of any patient into the study.

The Principal Investigator is responsible for informing the SERI Institutional Review Board and HSA (where applicable) of any amendments to the protocol or other study-related documents, as per local requirement.

## Funding and Insurance

|  |  |
| --- | --- |
| Funding will be provided under SNEC HREF grant  The usual trial indemnity for SERI applies, but no other insurance will be provided. |  |
|  |  |
|  |  |

## References

| 1. Bloch-Michel E, Dussaix E, et al. Possible role of Cytomegalovirus infection in the etiology of the Posner-Scholossmann syndrome. Int Ophthalmol 1987;11:95-96.  2. Markomichelakis NN, Canakis C, et al. Cytomegalovirus as a cause of anterior uveitis with sectoral iris atrophy. Ophthalmology 2002;109: 879-82.  3. De Schryver I, Rozenberg et al. Diagnosis and treatment of cytomegalovirus irodocyclitis without retinal necrosis. Br. J Ophthalmol 2006; 90: 852-5.  4. Van Boxtel LA, van der Lelij A et al. Cytomegalovirus as a cause of anterior uveitis in immunocompetent patients. Ophthalmology. 2007 Jul;114(7):1358-62.  5. Chee SP, Bascal K, Jap et al et al. Clinical features of cytomegalovirus anterior uveitis in Immunocompetent patients. Am J Ophthalmol 2008 May;145(5):769-71.  6. Chee SP, Bascal K, Jap et al. Corneal endotheliitis associated with evidence of cytomegalovirus infection. Ophthalmol 2007;114:798-803  7. Kawaguchi T, Sugita S, Shimizu N, et al. Kinetics of aqueous flare, intraocular pressure and virus-DNA copies in a patient with cytomegalovirus iridocyclitis without retinitis. Int Ophthalmol. 2007 Dec;27(6):383-6.  8. Yamauchi Y, Suzuki J, Sakai J, et al. A case of hypertensive keratouveitis with endotheliitis associated with cytomegalovirus. Ocul Immunol Inflamm. 2007 Sep-Oct;15(5):399-401  9. Koizumi N, Suzuki T et al. Cytomegalovirus as an etiologic factor in corneal endotheliitis. Ophthalmology. 2007 Jul 30;  10. Suzuki T, Hara Y Uno T et al. DNA of cytomegalovirus detected by PCR in aqueous of patient with corneal endotheliitis after penetrating keratoplasty. Cornea. 2007 Apr;26(3):370-2.  11. Teoh SB, Thean L, et al. Cytomegaolovirus in aetiology of Posner-Schlossman syndrome. Eye 2005;19 1338-1340.  12. Mietz H, Aisenbrey S, et al. Ganciclovir for the treatment of anterior uveitis. Graefes Arch Clin Exp Ophthalmol. 2000 Nov;238(11):905-9  13. Sira M, Murray PI. Treatment of cytomegalovirus anterior uveitis with oral valaciclovir. Ocul Immunol Inflamm. 2007 Jan-Feb;15(1):31-2  14. Chung RS, Chua CN. Intravitreal ganciclovir injections in aqueous cytomegalovirus DNA positive hypertensive iritis. Eye. 2006 Sep;20(9):1080.  15. Pouliquen P, Elena PP, Malecaze F, et al. Assessment of the safety and local pharmacokinetics of a 0.15% gel of ganciclovir (VIRGAN) in healthy volunteers.  16. Castela N, Vermeire N et al. Ganciclovir Ophthalmic gel in Herpes Simplex Virus Rabbit Keratitis: intraocular pentration and Efficacy. J Ocul Pharmacol. 1994 Summer;10(2):439-51.  17. Morlet N, Young S, et al. High dose intravitreal ganciclovir injection provides a prolonged therapeutic intraocular concentration. Br_J Ophthalmol 1996; 80: 214-216  18. VIRGAN 0.15% ophthalmic gel drug insert. |
| --- |
